# Nonpharmacological Complementary Interventions for the Management of Pain after Third Molar Surgery: An Umbrella Review of Current Meta-Analyses

**DOI:** 10.1155/2022/1816748

**Published:** 2022-10-26

**Authors:** Parsa Firoozi, Saulo Gabriel Moreira Falci, Seong-Gon Kim, Leon A. Assael

**Affiliations:** ^1^Student Research Committee, School of Dentistry, Zanjan University of Medical Sciences, Zanjan, Iran; ^2^Department of Dentistry, Oral and Maxillofacial Surgery Section, Federal University of Vales Do Jequitinhonha E Mucuri (UFVJM), Rua da Glória 187, Diamantina, Minas Gerais 39100-000, Brazil; ^3^Department of Oral and Maxillofacial Surgery, College of Dentistry, Gangneung-Wonju National University, Gangneung 28644, Republic of Korea; ^4^Department of Preventive and Restorative Dentistry, University of California, San Francisco, CA, USA; ^5^School of Dentistry, University of Minnesota, Minneapolis, MN, USA

## Abstract

**Objectives:**

To provide a nonbiased, complete assessment of what the evidence from meta-analyses informs us about complementary and nonpharmacological treatment options for the management of pain after third molar surgery, as well as highlight any discordancy, gaps, or lack of evidence among meta-analyses.

**Methods:**

The quality of the included systematic reviews was assessed using the ROBIS tool. Corrected covered area (CCA) was calculated for pairs of similar meta-analyses to identify the amount of overlap. Reviews that were the most recent, comprehensive, and had adequate quality were considered for analyses when reviews showed a high overlap. In cases with a low amount of overlap among meta-analyses, all eligible studies were included. Also, citation matrices were constructed to address overlap. A network meta-analytical approach was adopted to rank different interventions.

**Results:**

Ten meta-analyses were included for quantitative synthesis. The quantitative analysis revealed that platelet-rich fibrin and its derivatives as well as ozone therapy reduce early and late pain better than the other complementary interventions compared to control (no complementary intervention).

**Conclusions:**

Despite the shortcomings of included meta-analyses, consolidated evidence suggests that platelet-rich-fibrin and its derivatives as well as ozone therapy outperform the other nonpharmacological complementary interventions in reducing early and late postsurgical pain following third molar extraction. However, the results should be interpreted with caution due to an unclear risk of bias and lack of firm evidence in the included meta-analyses. Moreover, there is a need for a standard protocol for the application of nonpharmacological complementary interventions.

## 1. Introduction

The most frequently impacted tooth in the mandible is the third molar [1, 2]. Hence, third molar surgery is a common procedure for oral surgeons, and it is frequently linked with problems such as pain, edema, and trismus [3].

These sequelae are caused by postoperative inflammatory reactions, which may impede patients' everyday functions and compromise their quality of life throughout the recovery period [4]. To control postoperative inflammatory reactions, traditional allopathic analgesics are widely used [5].

On the other hand, nonpharmacological complementary interventions are proposed to enhance pain relief, and reduce analgesic use, mitigating the unwanted effects and contraindications of allopathic medications [6–12]. This goal is especially important in reducing or eliminating the use of opioid analgesics. Furthermore, nonpharmacologic methods may improve the outcomes of typical anti-inflammatory medications used after third molar surgery [8].

Several nonpharmacological complementary methods have been reported in evidence-based systematic reviews with or without meta-analyses for controlling postoperative morbidities related to third molar removal. They include the application of low-level laser therapy (LLLT) [13], cryotherapy [11], application of platelet-rich fibrin (PRF) [12], application of hyaluronic acid [10], ozone therapy [7], application of drainage [9], and kinesio taping (sports tapes) [8].

Systematic reviews have been increasing to such an extent in recent years that make a unified conclusion on the present state of clinical evidence that has emerged as an important area of inquiry. With the volume of reviews regarding the management of complications after third molar surgery, a meta-level synthesis is needed to make sense of the evidence from published systematic reviews. In addition, like with all forms of research, the quality of the systematic reviews already published may vary, and their conclusions may be flawed due to methodological weaknesses and biases.

Therefore, this umbrella review (overview of reviews) is intended to provide a nonbiased, complete assessment of what the evidence from systematic reviews with meta-analyses informs us about nonpharmacological complementary treatment options for the management of pain after third molar surgery. Additionally, it seeks to pinpoint any remaining research gaps and provide a list of suggestions for enhancing the quality of upcoming studies in this field.

Accordingly, the findings may give evidence that can be utilized to develop or update decision-making guidelines.

## 2. Materials and Methods

The current review of systematic reviews with meta-analyses was registered in PROSPERO (code : CRD42022326584). The Preferred Reporting Items for Systematic Reviews and Meta-Analyses (PRISMA) guideline (updated in 2020) was followed [14].

The research purposes for this umbrella review were the following items:

1. What do we currently know so far about nonpharmacological complementary interventions regarding the management of pain after third molar surgery based on the available meta-analyses?
2. Which nonpharmacological and complementary interventions are more effective in reducing pain after third molar surgery based on the available meta-analyses?
3. To quantitatively compare different nonpharmacological, complementary, and nonsurgical interventions for the management of pain after third molar surgery.
4. To highlight any discordancy among meta-analyses.
5. To critically appraise the available meta-analyses and provide a list of recommendations for enhancing the quality of future systematic reviews and clinical trials.

### 2.1. Eligibility Criteria

The PICO(S) structure was delineated as follows:

Population: patients who underwent third molar surgery.

Intervention: all available nonpharmacological complementary interventions for the management of pain after third molar surgery.

Comparator: no complementary intervention (or placebo).

Outcome: reduction of pain.

Study type: systematic reviews of RCTs with quantitative meta-analyses.

Exclusion criteria included qualitative systematic reviews without meta-analyses; RCTs; observational studies; case reports; conference papers; narrative and scoping reviews; letters to the editors; and animal studies. Studies that did not meet the inclusion criteria were excluded.

No restrictions regarding language or publication date were applied.

### 2.2. Search Strategy and Information Sources

A search of three electronic databases was carried out up to 20 June 2022: MEDLINE, Scopus, Web of Science, and Embase. As a grey literature source, the first 100 hits of Google Scholar were also reviewed. Using MeSH terms and related free keywords, a literature search was done in the abovementioned databases (Supplementary File 1). No restrictions regarding language and date of publication were applied. Furthermore, a hand search of the relevant journals and textbooks was conducted.

### 2.3. Data Selection and Collection Process

Duplicate records were eliminated when all entries were imported into the Mendeley software (version 1.19.8). To identify suitable meta-analyses, two researchers (PF and SGK) independently assessed the titles and abstracts of all retrieved data. The full texts of possibly eligible studies were then obtained and scrutinized by two independent reviewers (PF and SGK) using the predetermined inclusion criteria. Two authors (PF and SGK) extracted data from the final eligible studies individually using customized pilot-tested extraction forms. Any controversy between two reviewers during the study selection and data collection stages was handled by a discussion with a third reviewer (LAA) until a consensus was established.

### 2.4. Data Items

A modified Joanna Briggs Institute (JBI) data extraction form was used to obtain study characteristics [15]. This modified data extraction form included the following items: authors, year of publication, objectives, participants (characteristics/total number), description of the intervention, description of the comparator, sources searched, range (years) of included studies, number of studies included, appraisal tools used, appraisal rating, findings, significance, heterogeneity, and publication bias. For quantitative synthesis effect sizes, 95% CIs and the number of subjects in each arm were extracted.

### 2.5. Study Risk of Bias Assessment

The authors (PF and SGMF) independently assessed the quality of the selected systematic reviews using the ROBIS criteria [16]. The tool is comprised of 3 phases: (1) assess relevance (optional), (2) identify concerns with the review process, and (3) judge the risk of bias. Phase #2 covers four domains through, which bias may be introduced into a systematic review/meta-analysis:

1. Study eligibility criteria
2. Identification and selection of studies
3. Data collection and study appraisal
4. Synthesis and findings [16].

Studies with considerable weakness were rated as high risk of bias (3 to 4 negative points in phase #2), and those without considerable weakness (one negative point in phase #2) were rated as low risk of bias. Otherwise, those studies with 2 negative points in phase #2 were rated as “some concerns.”

### 2.6. Overlap Assessment

In cases where there was more than one systematic review for complementary treatment, to solve the potential overlapping issue, a citation matrix was created that showed which original clinical trials had been included in similar reviews. Utilizing the corrected covered area (CCA) [17], the overlap was measured quantitatively at the review level [18].

Predetermined overlap thresholds were used for the interpretation of overlap (0–5%, slight; 6–10%, moderate; 11–15%, high; >15%, very high) [17]. CCA calculations for pairs of systematic reviews were performed and presented as grids.

When reviews showed very high CCA, only one meta-analysis that had the highest quality according to the ROBIS tool was considered for the main analysis [16]. In cases with multiple high-quality reviews, the most recent one with more RCTs was selected.

On the other hand, reviews with a slight overlap and new relevant information were included in the sensitivity analysis. Additionally, as a sensitivity analysis, the main selected high-quality meta-analysis was also compared to a more recent meta-analysis with adequate quality including more recent RCTs with relevant information to test the robustness of the primary results. Low-quality meta-analyses were excluded from all analyses.

Moreover, Jadad's algorithm was adopted to solve any discordance among overlapped meta-analyses [19].

### 2.7. Data Synthesis

The methodological approach for data synthesis demonstrated in the Umbrella Review book was adopted [20]. Aggregated effect sizes (ESs) with 95% confidence intervals (CIs) for pain outcomes were extracted, and then, converted into a common effect estimate (standardized mean difference; SMD) and standard error (SE). Furthermore, for early postoperative pain, ESs corresponding to 48 hours and 72 hours after surgery were pooled into a single ES and variance, and for late pain, ESs corresponding 5 to 7 days after surgery were pooled into a single ES and variance (if applicable) [9].

Statistical methods regarding the application of the network meta-analysis model for an umbrella review were used. Network meta-analysis synthesizes evidence from individual studies (such as randomized controlled trials), while umbrella review synthesizes evidence from traditional pairwise meta-analyses to undertake multiple treatment comparisons [20]. Therefore, the frequentist network meta-analysis with the fixed-effect model was used to visually rank different complementary interventions [21]. For this purpose, the “netmeta” package and R software were used. In Cochrane's guide for systematic reviews and meta-analyses, Cohen offers a suggested rule of thumb for clinically interpreting SMD: an SMD of 0.2 is seen as a mild clinical effect, 0.5 as a moderate effect, and 0.8 as a substantial effect [22, 23]. The abovementioned rule was used to interpret the quantitative results.

## 3. Results

### 3.1. Identification of the Eligible Meta-Analyses

A total of 659 papers were retrieved from 4 main electronic databases and Google Scholar. Twenty-two eligible published meta-analyses were considered for inclusion in this umbrella review after titles and abstracts were screened and duplicate meta-analyses were removed [7, 9, 12]; do [6, 8, 10, 11].

After reading the full texts, three papers [24–26] were ruled out (due to lack of quantitative synthesis for pain outcome and not meeting the eligibility criteria).

**Figure 1:**
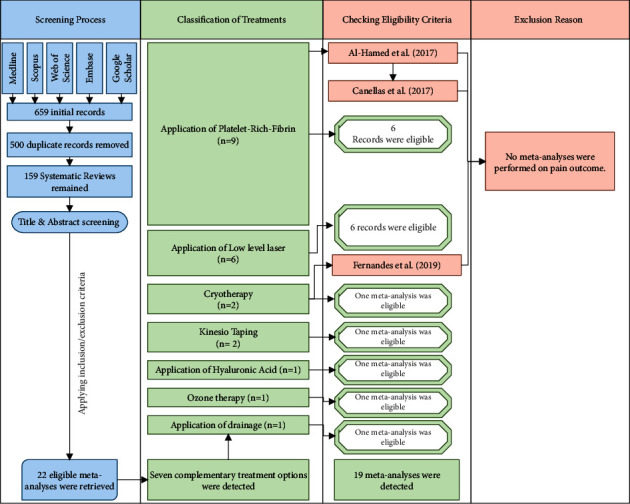
Diagram of the literature search and identification of potentially eligible meta-analyses.

Finally, 19 eligible meta-analyses were retrieved (Figure 1). Seven types of complementary interventions were identified among the retrieved meta-analyses including PRF application [12, 27–32], LLLT [33], [6, 34–37], Kinesio taping [8, 38], cryotherapy [11], surgical drainage [9], ozone therapy [7], and hyaluronic acid application [10].

### 3.2. Quality Assessment

**Figure 2:**
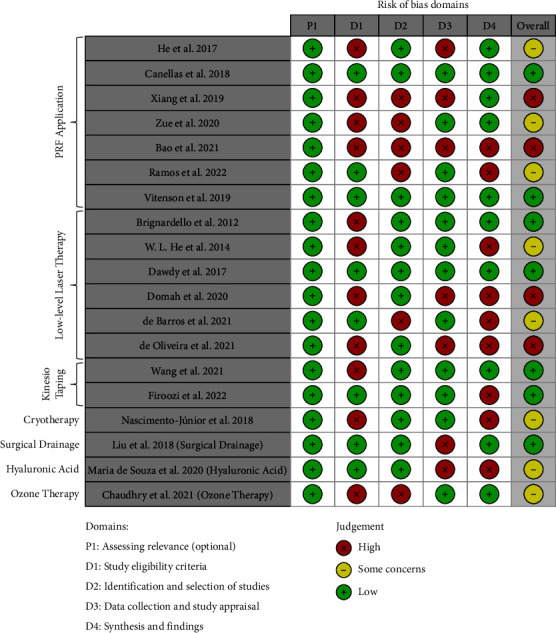
Risk of bias assessment.

All studies addressed the target review question appropriately (phase #1). Among 19 meta-analyses, 12 studies [12, 27, 29, 32] [33], [35–38]; [7, 11, 24] showed a high risk of bias regarding eligibility criteria (due to unclear restrictions regarding publication date or language and weakly described inclusion/exclusion criteria). Six studies [7, 12, 13, 27, 30, 32] had a high risk of bias regarding the study selection process (due to lack of specified databases' searched strategy or potential risk of selection of RCTs by only one reviewer instead of 2≤ independent reviewers). Seven studies [10]; [27, 29, 32, 35, 36, 39] had a high risk of bias regarding the data collection step (according to unclear efforts made to minimize errors in data collection and quality assessment). In terms of synthesis, 9 studies [10]; [8, 11, 27]; [13, 30, 35–37] showed a high risk of bias (due to lack of appropriate interpretation and justification of the observed high heterogeneity or due to lack of sensitivity analysis where possible) (Figure 2).

### 3.3. Overlap Assessment

The overall overlap (CCA) of the original RCTs for Kinesio taping, PRF application, and LLLT was 80%, 27.1%, and 24%, respectively, indicating a very high overlap. Two meta-analyses had low overlap and matched with other meta-analyses evaluating PRF application [27, 31]. One meta-analysis showed low overlap with other meta-analyses evaluating LLLT [33]. The other meta-analyses showed high to very high overlap in terms of included RCTs.

### 3.4. Study Selection Process

#### 3.4.1. PRF Application

**Figure 3:**
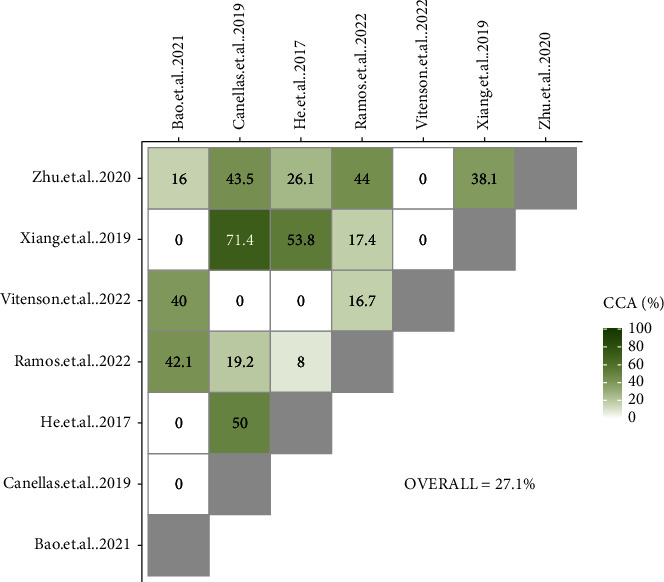
CCA grids of meta-analyses evaluating the effectiveness of PRF application in reducing pain after third molar surgery.

**Figure 4:**
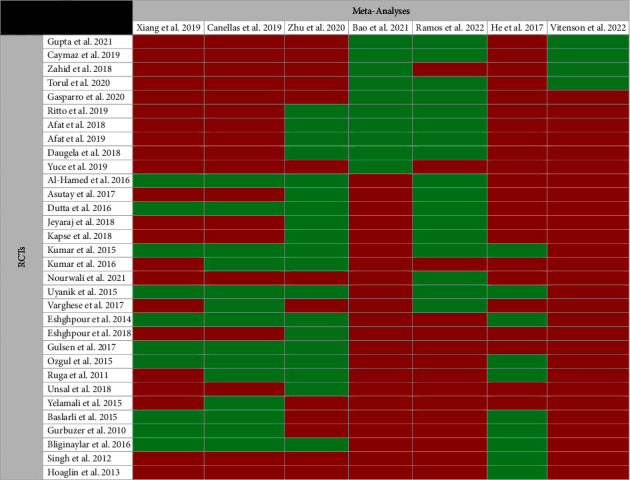
Citation matrix of meta-analyses evaluating the effectiveness of PRF application in reducing pain after third molar surgery (Green = included and Red = not included).

Since Canellas et al.'s study [28] showed the lowest risk of bias based on the ROBIS tool and had a very high overlap with other meta-analyses (due to evaluating any type of PRF with any preparation protocol), it was included in the primary quantitative analysis. The studies conducted by Bao et al. [27] and Xiang et al. [32] had a high risk of bias and were excluded. Among 3 other meta-analyses [12, 29, 30] with adequate quality, the study conducted by Ramos et al. [30] was the most recent and comprehensive (with more included RCTs), accordingly, was included in the sensitivity analysis to test the robustness of the results. Moreover, the study conducted by Vitenson et al. [31] had the lowest overlap with other meta-analyses (due to evaluating only new centrifugation protocols) and completely addressed our eligibility criteria. Therefore, it was included in addition to the other meta-analyses (Figures 3 and 4).

#### 3.4.2. LLLT

**Figure 5:**
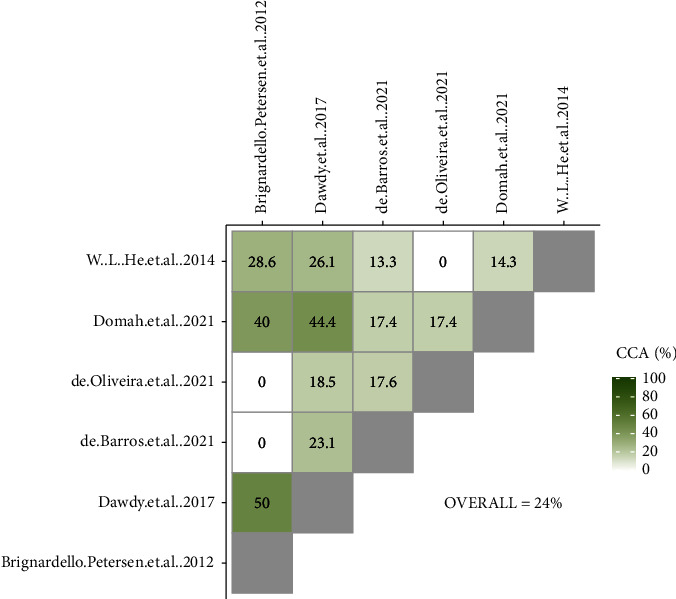
CCA grids of meta-analyses evaluating the effectiveness of low-level laser therapy in reducing pain after third molar surgery.

**Figure 6:**
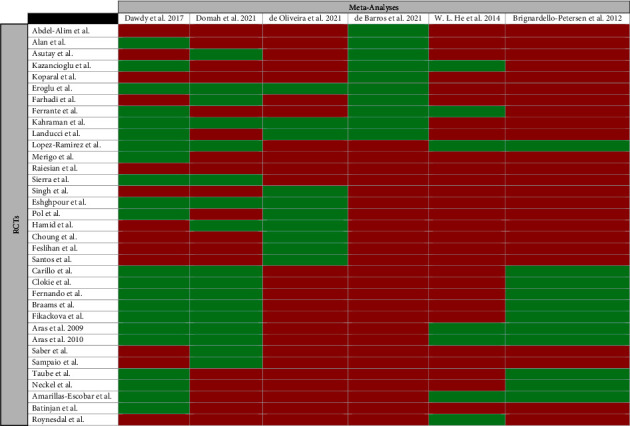
Citation matrix of meta-analyses evaluating the effectiveness of low-level laser therapy in reducing pain after third molar surgery (Green = included and Red = not included).

Brignardello-Petersen et al.'s [33] and Dawdy et al.'s [34] studies had the lowest risk of bias. Due to the lower overlap of Brignardello-Petersen et al.'s study [33] with other meta-analyses and outdated search strategies compared to Dawdy et al.‘s, Dawdy et al.'s study [34] was selected for the main analysis. Domah et al.'s [35] and de Oliveira et al.'s [36] studies were excluded from the analyses due to their high risk of bias. Among two remaining meta-analyses [6, 37] with moderate risk of bias and high overlap, the study conducted by de Barros et al. [13] was included in the sensitivity analysis due to the inclusion of more recent RCTs compared with Dawdy et al.'s study (Figures 5 and 6).

#### 3.4.3. Kinesio Taping

**Figure 7:**
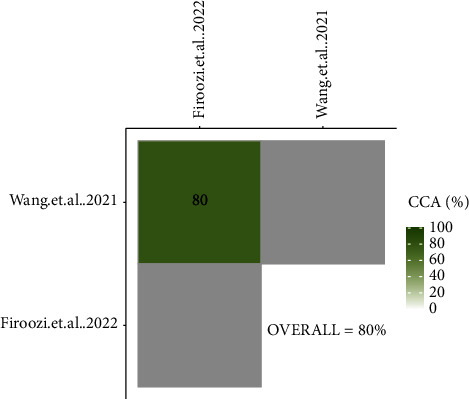
CCA grids of meta-analyses evaluating the effectiveness of Kinesio taping in reducing pain after third molar surgery.

**Figure 8:**
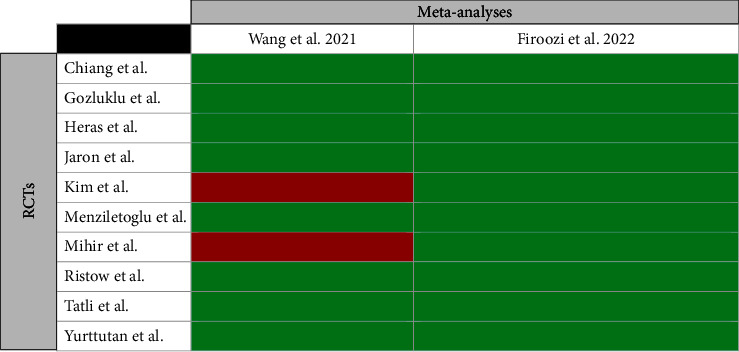
Citation matrix of meta-analyses evaluating the effectiveness of Kinesio taping in reducing pain after third molar surgery (Green = included and Red = not included).

According to an equal quality of two meta-analyses [8, 38] evaluating KT and a very high amount of overlap between them, the most recent one [8] with more trials was selected for the main analysis, and the other [38] with a lower number of included RCTs was excluded. Because there was no study with a slight overlap and newer information or moderate quality meta-analysis (with more recent trials), no sensitivity analysis for KT was applied (Figures 7 and 8).

### 3.5. Surgical Drainage, Ozone Therapy, Cryotherapy, and Application of Hyaluronic Acid

Only one meta-analysis was available for each of the abovementioned complementary interventions, and all were included in the main analysis [7, 10, 39].

### 3.6. Characteristics of Selected Meta-Analyses for Quantitative Synthesis

Ten meta-analyses were selected for quantitative synthesis which was published between 2018 and 2022. MEDLINE and CENTRAL were searched in all the included meta-analyses. The aggregate sample size varied from 132 to 1060 patients. Only two systematic reviews used Cochrane RoB tool-2 [8, 31] and others used the Cochrane RoB tool-1 [13]; [7, 9–12]. The total number of included RCTs ranged from 4 to 21. Two studies evaluated publication bias [9, 34] (Tables 1 and 2).

### 3.7. Analyses of the Quantitative Outcomes

Investigating only high-quality meta-analyses studies with minimal biases, a comparison of complementary interventions compared to control (no intervention) revealed that ozone therapy is the most effective complementary intervention (with a large clinical effect: SMD = −0.84; 95% CI [−1.09 ∼ −0.59]) and low-level laser therapy is the least effective (with a minimal clinical effect: SMD = −0.32; 95% CI [−0.49 ∼ −0.15]) complementary intervention in controlling early pain. Moreover, Kinesio taping, surgical drainage, and PRF application had a moderate clinical effect.

**Figure 9:**
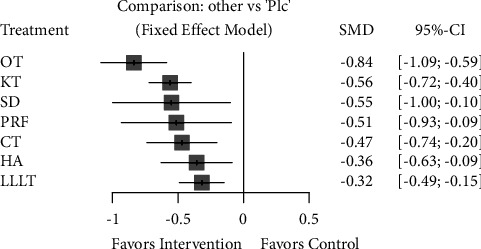
Forest plot comparing different complementary interventions for reducing early pain after third molar surgery. PRF: platelet-rich fibrin; SD: surgical drainage; CT: cryotherapy; HA: hyaluronic acid; KT: Kinesio taping; LLLT: low-level laser therapy; OT: ozone therapy; Plc: placebo.

**Figure 10:**
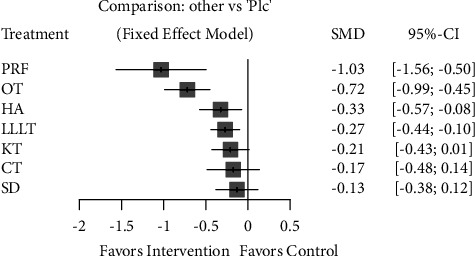
Forest plot comparing different complementary interventions for reducing late pain after third molar surgery. PRF: platelet-rich fibrin; SD: surgical drainage; CT: cryotherapy; HA: hyaluronic acid; KT: Kinesio taping; LLLT: low-level laser therapy; OT: ozone therapy; Plc: placebo.

On the other hand, PRF application (with a large clinical effect: SMD = −1.03; 95% CI [−1.56 ∼ −0.50]) and surgical drainage (with a small clinical effect: SMD = −0.13; 95% CI [−0.38 ∼ 0.12]) were the most and the least effective complementary interventions in controlling late pain, respectively. Ozone therapy showed upper moderate clinical effect in controlling late pain (SMD = −0.72; 95% CI [−0.99 ∼ −0.45]) (Figures 9 and 10).

### 3.8. Sensitivity Analysis

Considering the most recent meta-analyses with the lowest overlap, a comparison of complementary interventions compared to control (no intervention) revealed that the application of PRF derivatives, prepared with new protocols, and ozone therapy showed the largest clinical effect, and low-level laser therapy showed the least clinical effect in controlling early pain. Kinesio taping and surgical drainage had a moderate clinical effect in controlling early pain.

**Figure 11:**
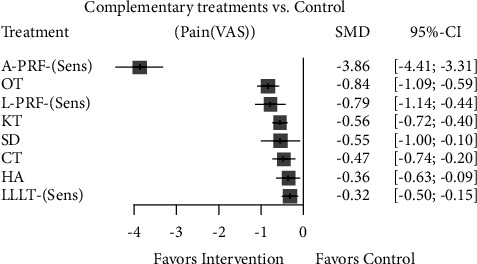
Forest plot comparing different complementary interventions for reducing early pain after third molar surgery after sensitivity analysis. A-PRF: advanced-platelet-rich fibrin; L-PRF: leukocyte-platelet-rich fibrin; SD: surgical drainage; CT: cryotherapy; HA: hyaluronic acid; KT: Kinesio taping; LLLT: low-level laser therapy; OT: ozone therapy; Plc: placebo.

**Figure 12:**
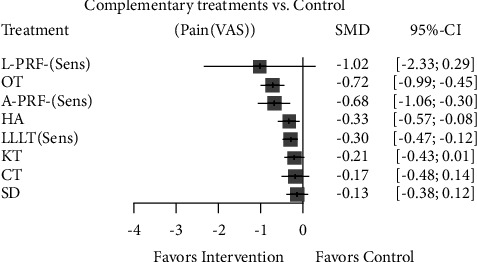
Forest plot comparing different complementary interventions for reducing late pain after third molar surgery after sensitivity analysis. PRF: platelet-rich fibrin; SD: surgical drainage; CT: cryotherapy; HA: hyaluronic acid; KT: Kinesio taping; LLLT: low-level laser therapy; OT: ozone therapy; Plc: placebo.

Similarly, PRF derivative application and ozone therapy were the most effective complementary interventions in controlling late pain. The other complementary interventions showed minimal clinical effectiveness in controlling late pain (Figures 11 and 12).

## 4. Discussion

Pain is a common side effect of dental surgical and nonsurgical extractions in the postoperative period, which gradually fades over time [40]. Pharmacological [41] and nonpharmacological [6–10]; [11, 12] approaches are proposed for alleviating pain after third molar surgery. To lessen the adverse effects and consumption of allopathic drugs, nonpharmacological supplementary therapies are recommended [8]. Hence, this umbrella review with integrated network meta-analysis aimed to provide a nonbiased, complete assessment of what the evidence from systematic reviews/meta-analyses informs us about complementary interventions for the management of pain after third molar surgery, as well as provide a list of recommendations for future primary and secondary studies.

### 4.1. Early Pain

The results of primary synthesis revealed that ozone therapy reduces early pain after third molar surgery better than the other complementary treatment options. Probably the mechanisms of action of ozone therapy involve the activation of antioxidant mechanisms. Moderate oxidative stress has been shown to activate nuclear transcription factors such as nuclear factor-erythroid 2-related factor 2 (Nrf2), hypoxia-inducible factor-1a (HIF-1a), nuclear factor of activated T cells (NFAT), and activated protein-1 (AT-1) [42]. Ozone, which is ten times more hydrosoluble than oxygen, quickly dissolves in the aqueous environment of plasma and is partially quenched by hydrophilic antioxidants such as reduced glutathione, ascorbic, and uric acids serving as sacrificial molecules (between 20% and 40%), while the majority reacts with polyunsaturated fatty acids (PUFA) carried by the albumin (60%) [42]. In terms of pain relief, ozone therapy outperformed prostacyclin [43]. When an oxygen/ozone combination is infiltrated, a highly oxidizing gas is infiltrated with an appropriate tissue diffusion capability. Medical ozone use leads to anti-inflammatory, analgesic, and antiedema results. Researchers also suggest that oxidizing algogenic receptors might block pain signals and activate the antinociceptive system [44]. Typically, ozone therapy is used in combining with usual therapies, whether systemic or local (infiltrations, applications of oils, and ozonated water) [45].

Despite the promising effects of ozone therapy on reducing early pain after third molar surgery, the results of the included meta-analysis were only based on a limited number of included RCTs with high heterogeneity in terms of methodology [7]. Accordingly, the recommendation of ozone therapy for clinical routine use requires firm evidence via conducting further high-quality RCTs with standardized methodology. Also, apparent is the lack of standard and safe protocol for ozone administration in oral surgery.

On the other hand, after performing a sensitivity analysis, new protocols of PRF preparation (classified into L-PRF and A-PRF) outperformed conventional PRF application. However, the results of the primary synthesis changed considerably after the sensitivity analysis. This shows that the way of PRF preparation (such as centrifuging speed and time) is a very important factor in the effectiveness of PRF application because it affects fibrin structure, the concentration of cytokines, growth factors, cells, and platelets [27, 46]. A-PRF application in the extraction socket performed better than the L-PRF in controlling early pain, and both were better than PRF at the beginning of the inflammatory process [30]. A-PRF has a higher proportion of monocytes than L-PRF, which allows for a faster vascularization of the area and a greater release of cytokines than PRF/L-PRF; these monocytes are crucial for the growth of blood vessels and bone regeneration. This may justify why A-PRF is better at controlling pain than L-PRF [47, 48].

Platelets become activated and begin releasing their products (platelet-specific proteins, non-platelet-specific proteins, calcium ions [Ca^++^], serotonin, cytokines, and growth factors) as soon as they come into contact with the test tube wall during PRF preparation. These platelet products, along with the glycan chain, are integrated into the fibrin matrix and play an important role in inflammation control. This is the likely mechanism of PRF in decreasing pain and the risk of infection and inflammation following third molar surgery [49]. Furthermore, PRF's stable fibrous architecture provides a three-dimensional scaffold for cytokines and growth factors, which contribute to leukocyte cell migration, hence, mediating the reduction of postoperative pain [50].

However, the application of PRF (especially L-PRF and A-PRF) still does not have a clear standard protocol per surgical procedure. For example, L-PRF has a quantity-dependent impact, but it is impossible to determine if one membrane would be sufficient for optimum efficacy in reducing pain [51]. Therefore, further studies are required to achieve a standard protocol for the intrasocket application of PRF/PRF derivatives. Controversial results among RCTs and a high amount of heterogeneity may result from the nonhomogeneous protocol of PRF preparation and application in oral surgery.

#### 4.1.1. Late Pain

The results of primary synthesis and sensitivity analysis showed the superiority of PRF application in controlling late pain. The progressively dissolving PRF also supports obviating surgical site debris, resulting in better late pain management with PRF [50]. Furthermore, the steady dissolving of PRF shields the extraction socket from the exterior environment, reducing the pathogenic bacterial load. After sensitivity analysis, L-PRF showed better results compared to A-PRF. This might be justified by L-PRF's denser structure, larger size, and slow-dissolving nature [52]. Besides, the results of the quantitative analysis showed significant results for Kinesio taping, surgical drainage, and cryotherapy for only early pain and not for late pain. This result was expected because these types of complementary interventions have the most effect on pain during the first three days after surgery when the inflammation increases.

### 4.2. Strengths and Limitations

The strength of the current umbrella review was a rigorous methodology for quantitative analysis by implementing network meta-analysis to rank different treatments. Besides, to the best of our knowledge, no study has compared different complementary interventions with each other; however, the present study has carried out this comparison using results from the network meta-analysis. Although all included systematic reviews were recent, it would be better if screening databases for newly published RCTs were performed. However, this procedure needs a lot of time to reanalyze previous meta-analyses with probable more recent RCTs [20].

## 5. Recommendations for Future Clinical Trials

Conducting head-to-head standard comparisons of different complementary interventions after oral surgery is recommended. Also, the aggregated effect of these complementary interventions should be investigated to determine if the results can be improved if they are used together.

Split-mouth design and small sample sizes of primary studies are criticized for some controversial and heterogeneous results. Some split-mouth designed trials have shown a null effect of complementary interventions [53–55]. In fact, split-mouth RCTs may lead to some bias since it may be difficult for patients to evaluate each surgical site independently as pain may irradiate to the opposite side. It is highly recommended that future trials consider this issue and wait for a minimum of seven days before the second surgery to make patients able to distinguish between the levels of pain on each surgical side [56]. Furthermore, investigators should consider the age of patients in future trials. In fact, the age of ≥25 years impacts healing and pain intensity and reduction [57].

Furthermore, the beneficial effect of complementary treatments might be affected by pre- or postoperative medications. Thus, it is highly recommended that future RCTs consider the standardization of analgesics and antibiotic administration for accurate comparisons. Conducting further high-quality studies with larger sample sizes focused on removing the effect of systemic analgesic effects both in the intervention and control groups is recommended. Therefore, performing comparative studies evaluating the efficacy of allopathic drugs versus nonpharmacological interventions will be indispensable.

Additionally, applying these nonpharmacological interventions in other oral surgical and nonsurgical operations seems necessary to be assessed in future studies.

## 6. Recommendations for Future Systematic Reviews and Meta-Analyses

It is recommended that meta-analytical studies apply subgroup analysis in terms of the design when there are both parallel design and split-mouth design RCTs. Also, it is recommended that future meta-analyses perform a sensitivity analysis when an outlier study is included. Furthermore, authors of systematic reviews are encouraged to check the quality assessment tools designed for systematic reviews when conducting meta-analyses.

The following items should also be considered when performing a meta-analysis for a better comparison of different complementary interventions:

1. The difficulty of third molar surgeries in treatment groups.
2. Medication protocol among the studies.
3. Type of anesthetics used in treatment groups.
4. Type of flap used in treatment groups.
5. Age of included participants in primary studies.
6. Clinical significance as well as statistical significance.

## 7. Conclusions

Based on the limited evidence, PRF and its derivatives as well as ozone therapy seem to be the best nonpharmacological complementary therapies to reduce early and late postsurgical pain after third molar extraction superior to other nonpharmacological approaches. However, the results should be interpreted with caution because of the unclear risk of bias in the included reviews and the lack of firm evidence in this regard. In addition, the need for a standard protocol for the application of different complementary therapies along with addressing optimized standard surgical interventions feels.

## Figures and Tables

**Table 1:** Characteristics of the included systematic reviews.

Authors		Complementary intervention	Participants	Intervention protocol	Comparator	Sources searched	Range of included studies (years)	Overall no. of included studies	Quality and certainty assessment tools	Appraisal rating	Measurement of pain	Findings	Sig.	Heterogeneity (I^2^)	Publication bias
Nascimento-Júnior et al.	[11]	CT	231 healthy patients with unilaterally or bilaterally impacted mandibular 3^rd^ molars	CT using ice packs was applied on the surgery side of the face for 5 to 30 minutes followed by resting periods during the first 24 h to 48 h after surgery	No CT	PubMed, WoS, Scopus, CENTRAL, ClinicalTrials.gov, OpenThesis, and GL	1985–2018	6	Cochrane RoB 1 tool + GRADE evidence certainty	RoB: considerable GRADE: low	VAS	Early: 48 h	N	0%	NR
												Early: 72 h	Y	0%	
												Late: day 7	N	62%	
Liu et al.	[9]	SD	409 healthy patients with fully or partially impacted mandibular 3^rd^ molars	SD using gauze, tube, or rubber drainage	No SD	PubMed, WoS, and CENTRAL	1988–2016	10	Cochrane RoB 1 tool	RoB: considerable	VAS	Early: 48 h–72 h	Y	0%	Not suspected
												Late: days 5–7	N	0%	Not suspected
Canellas et al.	[28]	PRF	514 healthy patients with unilaterally or bilaterally impacted mandibular 3^rd^ molars	Intrasocket application of PRF (protocol of centrifugation: 3000 rpm × 10 minutes)	No PRF application	PubMed, Embase, WoS, and CENTRAL + GL	2010–2017	13	Cochrane RoB 1 tool	RoB: moderate	VAS	Early: 72 h	Y	79%	NR
												Late: day 7	Y	0%	
Ramos et al.	[30]	PRF/l-PRF/A-PRF	510 healthy patients with unilaterally or bilaterally impacted mandibular 3^rd^ molars	Intrasocket application of PRF (protocol of centrifugation: 3000 rpm × 10 minutes/2700 rpm × 12 minutes)	No PRF application	PubMed, Embase, WoS, CENTRAL, and Virtual Health Library	2015–2021	17	Cochrane RoB 1 tool	RoB: moderate	VAS	Early: 72h	Y	68%	NR
												Late: day 7	N	92%	
Vitenson et al.	[31]	A-PRF	132 healthy patients with unilaterally or bilaterally impacted mandibular 3^rd^ molars	Intrasocket application of A-PRF (protocol of centrifugation: 1500 rpm × 14 minutes/1300 rpm × 13 minutes)	No PRF application/other biomaterials	MEDLINE Embase, Cochrane Library, and Scopus + GL	2019–2021	4	Cochrane RoB 2 tool + GRADE evidence certainty	RoB: low to moderate	VAS	Early: 48 h	Y	NA	NR
												Early: 72 h	Y	0%	
												Late: day 7	Y	31.04%	
Maria de Souza et al.	[10]	HAA	271 healthy patients with fully or partially impacted mandibular 3^rd^ molars	1-Hyaluronic acid gel: applied to the extraction socket (0.2 mL to 2 mL) 2-Hyaluronic acid spray: two puffs were applied to the extraction area	No HAA	CENTRAL, PubMed, Virtual Health Library, and WoS + GL	2015–2018	5	Cochrane RoB 1 tool + GRADE evidence certainty	RoB: moderate GRADE: very low (pain) low (trismus)	VAS	Early: 72 h	Y	0%	NR
												Late: day 7	Y	28%	
Chaudhry et al.	[7]	OT	173 healthy patients underwent 3^rd^ molar surgery	OT was applied using extraoral prob, ozonated water, or topical ozone gel	No OT	MEDLINE, CENTRAL, and Google Scholar	2013–2020	4	Cochrane RoB 1 tool + GRADE evidence certainty	RoB: Considerable GRADE: Moderate to low	VAS	Early: 72 h	Y	82%	NR
												Late: day 7	Y	79%	
Firoozi et al.	[8]	KT	444 healthy patients underwent 3^rd^ molar surgery	Applying KT on lymphatic nodes and ducts nearby surgical areas on the skin	No KT	CENTRAL, PubMed, Google Scholar, and Scopus + GL	2013–2021	9	Cochrane RoB 2 tool + GRADE evidence certainty	RoB: high (pain) to moderate (swelling and trismus) GRADE: moderate (pain) to low to very low (swelling) to moderate to high (trismus)	VAS	Early: 48 h	Y	53%	NR
												Early: 72 h	Y	81%	
												Late: day 7	N	98%	
Dawdy et al.	[34]	LLLT	1060 healthy subjects underwent 3^rd^ molar surgery	LLLT was applied intraorally, extraorally, or both	No LLLT/Sham	PubMed, Embase, CENTRAL, and ClinicalTrials.gov	1990–2017	21	Cochrane RoB 1 tool + GRADE evidence certainty	RoB: considerable Grade: low to moderate	VAS	Early pain 48 h	Y	95%	Not Suspected
												Late: day 7	Y	87%	
De Barros et al.	[6]	LLLT	648 healthy subjects underwent 3^rd^ molar surgery	LLLT was applied intraorally, extraorally, or both	No LLLT/Sham	PubMed, WoS, and CENTRAL	Up to July 2020	15	Cochrane RoB 1 tool	RoB: low	VAS	Early: 48 h	Y	21%	NR
												Late: day 7	Y	78%	

WoS: Web of Science; GL: grey literature; RCTs: randomized controlled trials; RoB: risk of bias; MD: mean difference; SMD: standardized mean difference; CT: cryotherapy; SD: surgical drainage; PRF: platelet-rich fibrin; L-PRF: leukocyte and platelet-rich fibrin; A-PRF: advanced platelet-rich fibrin; HAA: hyaluronic acid application; OT: ozone therapy; KT: Kinesio taping; LLLT: low-level laser therapy; VAS: visual analog scale; NR: not reported.

**Table 2:** Treatment effects of different complementary interventions.

Authors	Complementary intervention	Outcome	Method of measurement	TE	Lower limit	Upper limit	Intervention (no.)	Control (no.)	Overall SMD	Overall SE
Nascimento-Júnior et al.	CT	Early pain 48 h	MD	−0.72	−1.45	0.01	64	60	−0.470	0.136
		Early pain 72 h		−0.36	−0.59	−0.13	52	48		
Liu et al.	SD	Early pain	SMD	−0.55	−1.00	−0.10	135	134	−0.55	0.229
Canellas et al.	PRF	Early pain 48 h	SMD	−0.42	−1.04	0.21	75	75	−0.512	0.214
		Early pain 72 h		−0.59	−1.16	−0.02	141	141		
Ramos et al.	L−PRF	Early pain 72 h	MD	−1.07	−1.53	−0.60	67	67	−0.786	0.179
Vitenson et al.	A-PRF	Early pain 48 h	MD	−1.68	−1.889	−1.465	27	27	−3.86	0.279
		Early pain 72 h		−1.21	−1.342	−1.071	47	47		
Maria de Souza et al.	HAA	Early pain 72 h	MD	−0.68	−1.20	−0.17	104	108	−0.358	0.138
Chaudhry et al.	OT	Early pain 72 h	MD	−2.93	−3.77	−2.08	133	133	−0.837	0.128
Firoozi et al.	KT	Early pain 48 h	MD	−1.99	−2.68	−1.29	120	120	−0.560	0.082
		Early pain 72 h		−1.45	−2.10	−0.81	188	188		
Dawdy et al.	LLLT	Early pain	MD	−1.42	−2.18	−0.67	280	260	−0.318	0.087
De Barros et al.	LLLT	Early pain 48 h	MD	−0.59	−0.92	−0.27	240	245	−0.324	0.091
Nascimento−Júnior et al.	CT	Late pain	MD	−0.46	−1.28	0.37	82	78	−0.174	0.158
Liu et al.	SD	Late pain	SMD	−0.13	−0.38	0.12	168	167	−0.13	0.127
Canellas et al.	PRF	Late pain	SMD	−1.03	−1.58	−0.49	30	30	−1.03	0.272
Ramos et al.	L−PRF	Late pain	SMD	−1.02	−2.34	0.30	67	67	−1.02	0.667
Vitenson et al.	A−PRF	Late pain	MD	−1.89	−2.92	−0.86	57	57	−0.68	0.193
Maria de Souza et al.	HAA	Late pain	MD	−0.36	−0.64	−0.09	124	128	−0.325	0.127
Chaudhry et al.	OT	Late pain	MD	−1.22	−1.67	−0.78	113	113	−0.719	0.137
Firoozi et al.	KT	Late pain	MD	−1.25	−2.59	0.08	158	158	−0.207	0.113
Dawdy et al.	LLLT	Late pain	MD	−0.59	−0.96	−0.22	280	260	−0.27	0.087
De Barros et al.	LLLT	Late pain	MD	−0.76	−1.21	−0.32	252	255	−0.298	0.089

MD: mean difference; SMD: standardized mean difference; TE: treatment effect; SE: standard error.

## Supplementary Materials

Supplementary Materials Search strategy of electronic databases.
