# Supplementary material for: Nonpharmacological Complementary Interventions for the Management of Pain after Third Molar Surgery: An Umbrella Review of Current Meta-Analyses
Source: Pain Res Manag. 2022 Oct 26;2022:1816748. doi: 10.1155/2022/1816748 (PMC9629945; doi:10.1155/2022/1816748)
Supplement: Supplementary Materials — Search strategy of electronic databases. [file 1816748.f1.docx]

**Supplementary Table 1.** Search strategy of electronic databases.

| Database | Search String | Number of records |
| --- | --- | --- |
| MEDLINE | ((((((("Molar, Third"[Mesh]) OR third molar*[Title/Abstract]) OR molar*[Title/Abstract]) OR wisdom tooth[Title/Abstract]) OR wisdom teeth[Title/Abstract])))) AND (((((((Systematic review [Title/Abstract]) OR meta-analysis [Title/Abstract]) OR metaanalysis [Title/Abstract])))))) AND (sequelae OR sequels OR associated conditions OR coexistent conditions OR pain) | 241 |
| SCOPUS | (TITLE-ABS-KEY ((“Third molar” OR “third molars” OR “wisdom tooth” OR “wisdom teeth" ) ) AND TITLE-ABS-KEY ( ( "Systematic review" OR "meta-analysis" OR "metaanalysis" ) ) AND TITLE-ABS-KEY ( ( "sequelae" OR "sequels" OR "associated conditions" OR "coexistent conditions" OR "pain") ) ) | 94 |
| Web of Science | TS = ((third molar*) OR (wisdom teeth) OR (wisdom tooth)) AND TS = ((Systematic review) OR (meta-analysis)) AND TS = ((sequelae) OR (sequels) OR (associated conditions) OR (coexistent conditions) OR (pain)) | 129 |
| Embase | ('third molar'/exp OR 'third molar*':ab,ti OR 'wisdom teeth':ab,ti OR 'wisdom tooth':ab,ti ) AND ('systematic review'/exp OR 'meta-analysis'/exp OR ‘systematic review':ab,ti OR 'meta-analysis':ab,ti) AND (‘sequelae’:ab,ti OR ' sequels ':ab,ti OR ' complication* ':ab,ti OR ' pain ':ab,ti) | 195 |
| Overall number of hits | | 659 |
